# Medical and Undergraduate Student Perceptions on Scribing in an Emergency Department

**DOI:** 10.7759/cureus.13836

**Published:** 2021-03-11

**Authors:** Ilya Abelev, Jacqueline Fraser, Donaldo D Canales, Natasha Hanson, Paul Atkinson, David Lewis

**Affiliations:** 1 Emergency Medicine, Dalhousie University/Saint John Regional Hospital, Saint John, CAN; 2 Research Services, Horizon Health Network, Saint John, CAN; 3 Research, Saint John Regional Hospital/Horizon Health Network, Saint John, CAN

**Keywords:** scribe, research in emergency medicine

## Abstract

Background

A shift towards electronic medical records (EMR) has increased physician burnout and decreased physician satisfaction and productivity. One solution to alleviate EMR stressors is the implementation of medical scribes. Scribes have been shown to increase physician productivity and satisfaction. The study objective was to elucidate medical and undergraduate student scribing experience to determine if that experience can incentivize scribes to work in the emergency department.

Methods

Ten students scribed and shadowed at a tertiary ED between July 4, 2019, and August 10, 2019. Medical students participated in two scribing and two non-scribing (shadowing) sessions, each lasting four hours. Undergraduate students only had a scribing condition. To facilitate scribing, a laptop with a wireless keyboard was provided, as well as a stand-up laptop tray.

An exit survey and semi-structured interviews were conducted after the scribing experience. The majority of insights were extracted from interviews. Transcripts were coded into thematic coding trees and analyzed using thematic analysis.

Results

All undergraduate students preferred volunteering in the ED over other volunteer experiences. All undergraduates cited direct access to the medical field, resume building, and perceived value added to the health care team as motivators to continue scribing. Most students credited demystification of the medical profession as a motivator. Most medical students felt scribing should be integrated into their curriculum. Based on survey results, five undergraduate students would volunteer 40 hours/week.

Conclusion

Our study showed that a volunteer model of scribing is feasible. Importantly, scribing may be an invaluable experience for directing career goals and ensuring that students intrinsically interested in medicine pursue the profession. Although a volunteer model may not provide the desired benefit in terms of ED efficiency, it may be an integral part of training the next wave of physicians.

## Introduction

In the modern era of emergency department overcrowding, EDs are looking at ways to decrease patient length of stay and increase physician productivity. Also, the province of New Brunswick (NB) is shifting towards a centralized electronic medical record (EMR). EMR’s have been reported to increase physician stress, decreased satisfaction, and increase burnout [1]. Medical scribes have been shown to alleviate some of these stressors, and therefore improve physician well-being [2,3]. The introduction of EMR in the ED, particularly in the implementation stage, may increase physician load and decrease the efficiency with which physicians see patients [4]. 

In the United States and some parts of Canada, scribes are compensated out of the physician’s earnings. However, this cost to the physician is offset by the increased revenue earned from seeing more patients in a fee-for-service physician payment model [3-6]. In the majority of Canadian ED's, physicians are paid an hourly rate for clinical services provided that does not fluctuate with efficiency, so a salaried scribing model may not be feasible. The goal of the current study to determine if a volunteer model for scribing may be implemented where scribes work in the Saint John Regional Hospital (SJRH) ED in exchange for the experience.

## Materials and methods

Methods

In this study, the goal was to determine the experiences of both undergraduate and medical student scribes to be able to compare and contrast their experiences. Selecting different groups allowed us to gain insight into how the volunteering motivations for students differed before and after acceptance into medical school.

Participants

Convenience sampling was used to recruit undergraduate students (n=5) and medical students (n=5). All students provided written consent for participation. No incentives were given. Research ethics was obtained from the Horizon Health Research Ethics Committee (file # 100242). 

Study participants included medical and undergraduate students. Inclusion criteria for both groups was a typing speed greater than 50 words per minute determined based on qualifications for scribes of America [7]. Typing speed was measured through an online typing test simulator at typingtest.com [8]. The inclusion criterion for undergraduate students is that they are, at minimum, in their second year of university.

Intervention

Medical students participated in two scribing and two non-scribing sessions, each lasting four hours. The time period over which the shifts occurred was variable for each student based on availability. Potential order effects were minimized through the randomization of scribing and shadowing session order. That is, there were two scribings and two non-scribing conditions across four sessions for each student, making six possible sequences. Students were randomly assigned to one of the six sequences.

Survey and interview questions for undergraduate volunteers were informed by previous studies and local expertise [5,9,10]. Exit interviews and surveys were conducted immediately after the students completed their experience. Interviews followed a script to ensure consistency. Interview answers were recorded on voice recorders. De-identified files from the voice recorders were downloaded onto a secure laptop and uploaded to NVivo software for transcription and analysis.

Study Design and Time Period

Students scribed and shadowed between July 4, 2019, and August 10, 2019. Scribes followed physicians into patient rooms with a portable laptop tray after consent was gained from the patient. Scribes would use a template to transcribe relevant parts of the physician-patient interaction. At the end of the interaction, the physician would review the scribe's note and sign off on it. If time permitted, there would also be medical teaching moments for the scribe. When the scribe was not following the physician, they would wait by the cart until prompted to follow.

Materials

To facilitate scribing, a laptop with a wireless keyboard was provided, as well as a stand-up laptop tray. All notes were entered into the hospital’s EMR software. Scribes were also provided with medical scrubs that they were required to return at the end of each shift.

Training

To enhance data quality, prior to the beginning of the study, scribes underwent a standardized training program (Appendix 1). This training included but was not limited to orienting students to the ED, explaining their role as scribe, and teaching etiquette and technical scribing skills.

Study Setting

This study was conducted at the Saint John Regional Hospital, which is a tertiary center that has approximately 60,000 ED visits annually. The scribe study was carried out in the ED. Surveys were conducted at the adjacent medical school facility (neutral location) to avoid bias from conducting interviews in a hospital setting. 

Data Analysis and Quality Enhancement

Multiple individuals including a resident physician, medical student, research coordinator, and a qualitative research expert reviewed the data and coding on NVivo to limit bias.

After data were coded in NVivo, ideas and concepts were highlighted. Thematic analysis was used to analyze the data because of its flexibility [11]. The thematic analysis followed the following steps: familiarization with the data, generation of initial codes in NVivo, search for themes, review of themes, defining and naming themes, and finally producing the report [11].

## Results

A total of five medical students (four female, one male, mean age =24 years) and five undergraduate students (two female, three males, mean age = 19.2 years) participated in the study. All 10 students completed pre-study surveys, post-study surveys, and interviews. Undergraduate interviews yielded a total of 110 minutes of interview time (mean = 18.3 minutes) while medical student interviews totaled 129 minutes (mean = 21.51 minutes). 

Survey results and themes

After completion of the study, students completed an online survey. The majority of undergraduate students reported they would continue volunteering an average of four to eight hours a week in the ED if given the opportunity. Therefore, if the five students all volunteered in the higher end of the four to eight hours range (eight hours), they would only volunteer 40 hours a week (5 students x 8 hours/week = 40 hours/week). Thus, five students would only cover one full-time physician.

For a list of all themes and relevant quotes refer to Appendix 2. Specific themes are elaborated on in the "shared themes," "undergraduate student themes," and "medical student themes" below.

Shared themes

*Learning Experience* 

A few medical students described exposure to the ED as being a rare and unique opportunity for undergraduate students. This quote exemplifies the challenge of getting exposure to the ED and how valuable medical students perceived the learning experience to be.

Medical student: “Unless you know someone who works at the hospital and can get [you] in to shadow them… it'd be difficult to find those opportunities on your own. So I think it'd be good for a pre-med student just because it gives some initial exposure to the medical field.” This perspective was echoed by the undergraduate students.

Undergraduate student: “before the study I'd never been in the ED. And I thought that was really cool just to see how everything works and being exposed to that is definitely a major learning experience.”

Perceived value to health care team

Students either described themselves as being liabilities or assets to the health care team. The majority of medical and undergraduate students felt the feeling of being in the way detracted from their scribing experience. This feeling occurred when students did not have a role in the ED. For all students, scribing only occurred during the physician-patient interaction, leaving time periods where students would stand by their scribing cart, not having a role. It was during this time that students felt out of place. Additionally, medical students felt it was unclear when they were needed, and when they were getting the way.

Medical student: “[during shadowing] you're expected to kind of follow around for all the things the doctor is doing, whereas with scribing you're only really expected to be in the room with the doctor when they go see the patient. And so I'd be following around with my cart everywhere even when I didn't need to be and I just felt annoying.” However, most students felt they improved the efficiency in the ED through improved legibility of typed notes over physician handwritten notes.

Undergraduate student: “[Physicians] were very appreciative of [the scribing]. And they said multiple times how helpful they thought it would be because then other doctors would be able to read the note.”

Undergraduate student themes

*Learning Experience* 

The learning experience was a theme discussed by all undergraduate students. Students described their learning experience in terms of the mentorship they received from the physician, clinical teaching moments, exposure to triggers, and the knowledge they acquired and reinforced from their undergraduate education. They also spoke about the value of being walked through the thought process of a differential diagnosis, developing their professionalism, and improving both their verbal and non-verbal communication skills.

Undergraduate student: “When [the physician] was working with residents or things like that, I get to listen and when she was explaining things to the resident, it was also [like] she was explaining things to me … I could relate in terms of how she was explaining her train of thought and how she would use her problem-solving skills to teach the resident.”

Perceived Value to Health Care team

Perceived value to the health care team was a concern for all undergraduate students. Perception of their value was brought up in terms being a liability or an asset to the team. Students believed they were liabilities because of their slow typing speed, difficulty understanding the physician, poor spelling, lack of knowledge of medical terminology, and because of direct feedback from physicians on how scribing decreased efficiency in terms of patients seen. There was much less discussion on perceived value to health care team; one student described an interaction with the physician that they were most useful during the psychiatric consult. 

Undergraduate student: “I think [the physician] thought for some of [the patients] - just because of the short time - it was a little inefficient because I think she could've done it faster than I could've. Especially after we would leave the examination room and she'd just look over the things, and we'd add things, and we'd discuss a little more about what happened.”

*Increased Motivation to Pursue Medicine, Work Harder in School* 

All students believed their experience as a scribe would be a motivator for them to pursue academic and professional excellence. Students consistently perceived their experience in the ED as motivating them towards pursuing their medical career, in one case to the extent of writing the Medical College Admissions Test (MCAT). Reasons for increased motivation included enjoyment and excitement of being in the ED, and the demystification of what physicians do.

Undergraduate student: “It definitely motivates me to be in medicine more. Because before …I had no clue. Now I actually have an idea what it's like [to be a physician] ... it seems like I actually have a proper goal. Rather than I'm just going towards this thing that I’m just told is a great thing. Which generally isn't a good way to go. So yeah, I think it helped a lot.”

Medical School Application Strengthened 

All undergraduates believed their medical school application was improved because of the scribing experience. Students believed character-building experiences such as overcoming adversity and building empathy were valuable in making them more competitive in the application process. Additionally, having the experience on their resume was believed to strengthen their application.

Undergraduate student: “I think that [scribing will] be something that they'll see on my resume that I’ve written when I apply and they'll be like 'Oh.. what.. .no way!'”

Scribing (Referred to All Other Volunteer Experience)

All students preferred scribing to other volunteer experiences. Students preferred scribing because of the direct exposure to the medical field, better learning experience, closer match to their career aspirations, and more exciting volunteering experience. Additionally, one student believed they provided more value (tying into the perceived value theme) scribing than in their past experiences.

Undergraduate student: “Well, I volunteered before at the hospital. But I found the volunteer capacities they have there are more... bring a patient somewhere, answer some questions that people have. It's not really direct exposure to what doctors are doing. It's more clerical work almost. And, like, that is good to know. Because you are going to have to do that at some point. But it's not the direct exposure that I wanted. Any volunteer things I've done haven't been direct exposure so far. So, this was awesome.”

Medical student themes

*Learning experience* 

All medical students brought up their learning experience as a theme during the interview. However, medical students, compared to undergraduates, placed a greater focus on the value scribing provided to them compared to the value they provided to the physicians. When discussing the learning value of scribing, medical students believed it improved their charting skills. They also believed that scribing was a complementary experience to shadowing because it forced them to focus on pertinent negatives, improved their competence coming up with differentials, improved their ability to direct interviews, and their history-taking skills. One student brought up the I3 training provided as valuable before they are exposed to the software as clerks.

Medical student: “Scribing in the [ED] is a really good fast way to learn how to come up with a case writeup that's a little less formal than what you'd actually be doing in the [ED]. So, I would not be opposed to it being integrated into the curriculum.”

*Shadowing Preferred* 

Most medical students preferred shadowing over scribing. Shadowing was preferred because they believed the teaching moments were more valuable, physicians treated them more like students (as opposed to administrative staff), and there was a better quality of feedback.

Medical student: “I think I preferred the shadowing just because there was more teaching involved. So, they would teach me a lot more about what conditions were going on - their thought processes. Whereas the scribing was more just, keeping up with what they were doing. And not so much the communication about what was going on with the patient. Like, yeah. Like the teaching part.” 

## Discussion

“Perceived value to healthcare team” was a theme for both medical and undergraduate students that can be used to illustrate differences in how the two groups perceived the experience. The perception of value was based on feedback from ED staff and the objective increased legibility of typed notes compared to handwritten notes. During the interviews, undergrads discussed their perceived value to the team with significantly higher frequency (mentioned 27 times) compared to medical students (mentioned 4 times). Thus, undergrads appeared to have a lot more concern with the value they brought to the ED. In contrast, medical students were more concerned about the learning experience or the value that the project provided to them.

The medical students did not believe they developed their procedural skills throughout the course of the shadowing experience. Lack of skill development may be related to the short period of time participants spent shadowing. Two medical students mentioned that the shadowing portion of the study should have been longer to better develop clinical skills. Partly because of the short length of the study, one student did not have an opportunity to conduct their own histories and physicals, preventing them from meeting their learning goals; this was the only student who did not find the scribing experience as complementary to their education. Overall, the shadowing elective was favored among medical students over scribing. The motivation for participation for medical students was to develop skills and knowledge. Since there were better perceived teaching moments during the shadowing compared to scribing experiences, their learning goals were better met during shadowing. Although shadowing was preferred, most medical students believed that scribing should be integrated into the curriculum. The rationale for integration was that scribing provided a complementary learning experience. Specifically, scribing allowed students to gain insight into how to interact with the patient and glean medical pearls from observing the physician. Afterward, the students could apply this knowledge during the history-taking component of shadowing. 

Undergraduates preferred scribing over other volunteer experiences. Undergraduate preference for scribing was related to their direct access to the medical field, the demystification of the medical profession, resume building, and perceived value added to the health care team as their main motivations. However, based on 40 hours/week of volunteer time available across five students, scheduling and turnover limitations may make it difficult to have trained scribes available at all, or even peak times in the ED under a volunteer model. 

Scribes shifted their motivation to pursue medicine from extrinsic to intrinsic as a result of the experience. Before scribing, students described motivations as being extrinsic, e.g., Parental or societal pressures [12]. Student extrinsic motivation has been linked to poor study habits in medical school and exhaustion [13]. Exhaustion can lead to burnout and eventual dropout [14]. As a result of their scribing experience, students described their new motivation as being intrinsic. In this way, a scribing program may be beneficial for students' mental health as they decide whether to pursue medicine. Thus, a volunteer scribing program may be beneficial to help undergraduate students direct their career goals. Scribing may also provide an avenue where students from less privileged backgrounds - who do not have connections to medical doctors - to gain direct medical exposure. Thus, exposure to practicing physicians demystifies the profession and provides clarity in terms of medicine as a career path. However, students from less affluent backgrounds may not be able to afford to volunteer their time due to the high opportunity cost. Thus, the scribing program should be implemented in a way to mitigate selection bias towards higher family income students. 

Only one previous study examined student scribing experiences [5]. Lowry’s (2017) paper was methodologically different because it sampled only medical students who scribed before medical school, whereas this study investigates both medical and undergraduate student experiences immediately after scribing [5]. The findings of both studies were consistent: students were able to gain informed commitment to the profession through exposure to medicine and increased confidence to apply to medical school, both through an improved resume and personal development. Additionally, students were able to use the experience as a framework for learning through clinical teaching moments, observing doctor-patient interactions, and gaining clinical knowledge. 

Undergraduate students were concerned that they were slowing their physicians down. In one case, the physician informed their scribe that they were more efficient on their own. This decreased efficiency may be due to a lack of scribe training on medical terminology and an inadequate amount of time to develop the scribe-physician working relationship. At our ED, scribes received a pilot training program and were interviewed after 16 hours of scribing. However, research at the Queensway-Carleton Hospital that trained scribes using a professional service and included a two-month acclimation period found an efficiency benefit to using scribes [15]. Therefore, improved training and time spent with the physician may increase the efficiency benefit of scribes.

Limitations

Mandatory project participation for summer electives may have been a source of bias for one medical student. Another limitation of the study was that physicians who were assigned scribes were also responsible for teaching residents. Participants who were placed on shifts where physicians were responsible for residents may have had less involved teaching moments than physicians who did not have a resident. One medical student stated they were not able to make their own histories, preventing them from accomplishing their learning goals. Future scribing for medical students should include more open communication of student learning goals with physicians.

Clinical implications

Undergraduate students come with drawbacks as a volunteer workforce. There is significant training required for each student, a limited number of hours that students can volunteer, and there is a large turnover among students as they travel. Additionally, spelling and comfort with terminology were brought up as concerns by most undergraduate students as a factor that limited their efficiency benefit to physicians. One possible solution to knowledge and time-commitment problems is to integrate the scribing program with the university as an elective course, including a training and scribing component. However, there appeared to be a trade-off where the more time the physician spent providing an educational experience to the scribe, the less efficient benefits the scribe provided to the ED. For this reason, it is unclear what effect volunteer scribes have on ED efficiency and physician well-being. Regardless, the impact that the scribing experience has on training future physicians may be enough to justify the project’s implementation.

Research implications

Future research should look at the efficacy of different training protocols on scribe experience and expand the role of the participant to more than just scribing. Testing different training protocols is critical in assessing the value this model brings to the ED. Additionally, undergraduate scribes described their feeling of being out of place in the ED as the major drawback to this project. Thus, future studies on scribe experience should expand the scope of participants' responsibilities beyond scribing and assess how this impacts experience. Future studies should also investigate the benefit the volunteer scribe has on the ED with and without additional training.

## Conclusions

A volunteer model of scribing is feasible. However, scribe availability, potentially high scribe turnover, and limited time to develop a rapport with their physician may impact the efficiency benefit scribes provide to the ED. Further research needs to be conducted to evaluate scribe impacts on ED efficiency using a volunteer model. Importantly, scribing may be an invaluable experience for directing career goals and ensuring that students intrinsically interested in medicine pursue the profession. Medical students believed that scribing should be integrated into the curriculum. However, these students suggested scribing be added in their first year before they start taking their own histories to help them develop a framework for how to manage patients. While scribing provides significant benefits to students such as selecting and preparing the next wave of physicians, a volunteer model may not provide the desired benefit in terms of ED efficiency. 

## Appendices

Appendix 1

SJRHEM Scribe Etiquette - For Scribes

Dress code: Scrubs will be provided

Start shift: Scribe shift will be four hrs long (+/- 30mins); arrive 10 minutes before shift; change in learner’s office (need access); bring scribe laptop station to shift location (Raz or Acute); meet staff physician in Raz or Acute

Introductions

Shift coordinator: the nurse in charge of the department.

Ward clerk: the secretary/admin for the department - takes calls, arranges appointments, pages consultants.

Staff emergency physicians: Other emergency physicians on the shift.

Equipment: Pen and notepad.

Scribe computer: This will be kept in the learner’s office. Ensure it is plugged in and charging at the end of your shift.

Main Task

Scribing: Documenting the clinical encounter between the patient and physician. This involves listening to the questions the physician asks and documenting the patient's answer. Utilize the various template shortcuts to prepopulate the document. Paraphrase/abbreviate both questions and answers in order to keep up with dialogue. but try to keep the essence of the dialogue as accurate as possible

The physician may skip between some of the sections, e.g., history of presenting illness and past medical history. Get used to scrolling between these sections and entering the text under the correct heading.

Don’t guess any answers: If note sure - mark with a? and ask the physician at end of the encounter. While scribing has a machine at the foot end of the bed so that you can see the physician and the patient and are able to watch the clinical examination.

The physician will dictate the physical examination findings when the patient encounter has completed, leave the room with the physician. At this point, the physician will either move onto the next patient or return to the base station to enter "orders," etc.

If the physician moves onto the next patient, you should save the "Physician Clinic Note" and open up a new "Physician Clinic Note" for the next patient. Don’t worry, if the previous note is not perfect, it can be edited later. Once the physician has returned to the base station, open the relevant Physician Clinic Note(s) for editing. Correct any obvious spelling mistakes and use this opportunity to ask the physician to clarify any areas of content that you were unsure of.

The physician will then review the note and make corrections if required; if the patient has been discharged, the physician will enter the diagnosis and then ask you to print the document; ensure you select the correct printer (see scribe manual); collect the printed note and staple it together; give the note to the physician for filing with the chart.

Other Tasks

Photocopying - medication lists, letters, etc; fetching and returning the physician chart to the chart racks; communicating messages with ward clerk/secretary; ultrasound machine; entering patient details; moving to the patient bedside; cleaning the machine after use, etc.

What to Do When Not Involved in a Task?

Stand by at scribe laptop station; keep out of the way of nurses and other physicians but close to your physician; wait to be asked to do a task; "DON’T" use your cellphone for social/games, etc.

SJRH Scribing Instructional Manual

Teaching: These are not formal teaching sessions; however, observe the approach to clinical history taking and clinical examination, ask the physician to clarify points in the history and physical; the physician may have time to provide some teaching at certain points during the shift; "MAKE SURE TO PLUG IN LAPTOP WHEN NOT IN USE."

Open AllScripts (software where you will be scribing)

3. Physician will log in to the program using physician account

4. Find Patient name under ED Status Board

5. Select Enter Document

6. Select “Physician”. Then select “Physician Clinic Note”

7. Use an appropriate shortcode to generate a template

   · E.g., `gen

8. Scribe doctor-patient interaction

   · Consider other shortcodes when scribing (e.g., `abp and `pai) for abdominal pain-related symptoms and pain history, respectively.

9. Review scribed document with the physician

   · Physician will type in “diagnosis”

10. Save the document

   · You must select “physician clinic note” to be able to save the document

11. Go to the documents tab of Allscripts

12. Double click Physician Clinic Note of patient

13. Print out a physician note

14. ******MAKE SURE CORRECT PRINTER IS SELECTED********

After the initial assessment of the patient, the physician may order additional tests before discharging/ admitting the patient. If this occurs, do not print the physician's note until the physician instructs you to do so. Additionally, if the note needs to be modified, modifications can be done if modify is selected (tab found above print)

Other notes: Pick up scrubs for scribing; demonstrate ultrasound (locations and how to set up); how to clean ultrasound.

Appendix 2

**Table 1:** Themes and subthemes (students discussing theme/total students in group)

Shared Themes	Supporting Quote
Learning Experience (7/10)	
Medical Student	
exposure to the emergency room beneficial for undergraduates (2/5)	Well I think it's a good experience for pre-med students because they would otherwise really have limited opportunities to get inside a clinical setting. And to watch consultations. Ummm because normally those opportunities are ummm more or less reserved for medical students. Unless you, you know, know someone who works at the hospital and can, you know, get in to shadow them. But if you don't then I think it'd be difficult to find those opportunities on your own. So I think it'd be good for a pre-med student just because it gives some initial exposure to the medical field
Undergraduate Student	
exposure to the emergency room beneficial (5/5)	“being in the ER in that sense. Like before the study I'd never been in the ER. And I thought that was really cool just to see how everything works and being exposed to that is definitely a major learning experience.”
Perceived value to health care team	
Liability	
Feeling of being in the way (6/10)	you're expected to kind of follow around for all the things the doctor is doing, whereas with scribing you're only really expected to be in the room with the doctor when they go see the patient. And so I'd be following around with my cart everywhere's even when I didn't need to be and I just felt annoying.
Asset	
increased legibility of typed notes improving efficiency in the emergency department (8/10)	[Physicians] were very appreciative of it. And umm they said multiple times how helpful they thought it would be because then other doctors would be able to read the note. OR the secretaries were like. Oh this is going to be so much better because I'm actually gonna be able to read what the clinic note is saying. So that is obviously like an upside of the scribe thing. Ummm Cause i'd also noticed myself when I saw other sheets from other physicians that like or like even just the triage notes. When I was given it to kind of look over like the ummm the vital signs and stuff I could barely understand what it was even saying on there and sooo... putting that into typed format is definitely gonna be... I definitely believe would be a lot better and you can also quicken up things because like sometimes you might need to like ask the person who wrote it for confirmation. Is this what you said? which could be a long process if they're all the way in another department somewhere. You need to like go find them and call them up and they're not answering. Because you can't just go... this is what I think it is so I'm going to give them this and then oops uh the wrong thing. Ya that's one thing. Uhhh the, the health care team mentioned... nothing ever negative mentioned but I noticed after we would come out ummm the physician would like type like it wasn't like I did it and then it was done. It was like the physician needed to go on it again. And then type in more technical things that he hadn't have mentioned inside the thing or like spelling for some of the words. And so that took up extra time. Ummm but yeah. That's one thing I noticed. OR a couple nurses had said that they really found the whole project really helpful for them. Cause sometimes they can't read the physicians notes. So, like “this is so helpful. We can just see everything on the computer” so... I think that was really cool.
Undergraduate Student Themes	
Learning Experience (5/5)	
Mentorship from physician (1/5)	When [the physician] was working with residents or things like that, I get to listen and when she was explaining things to the resident, it was also knowledge she was explaining things to me not specifically but I could relate in terms of how she was explaining her train of thought and how she would use her problem solving skills to teach the resident but I also felt like it was a teaching opportunity for me as well.
Clinical teaching moments (3/5)	He helped me with my... spelling. Learning about the patient. Learning about the interactions. Like, I'd ask him questions about different courses of treatment. Different ailments. Like, “is this common? Is that common?" like "what are we going to do now". That kind of thing. I thought that was really interesting. …. It was good because I need the exposure now if I'm going to do it in the future. Because I thought I was fine but... turns out I wasn't. Now I need more exposure
Exposure to Triggers (2/5)	Ya.. so it was the first shift. I felt really faint watching a stapling up of a cut. So I left the room and then just recovered a little. And then for the rest of the scribing experience I tried my best to stay in the room and watch all the gross stuff so I have exposure. The shift I had yesterday, so my last one. We had a cut where the skin was falling off the finger and everything and I didn't even mind because it wasn't nearly as bad as the first thing so I found it really.... kind of just exposed me enough that I was capable of staying in the room the next time.
Knowledge Acquisition and Reinforcement (3/5)	Umm there was some stuff that I'd learned in school. That I recognized from like, that was being used in this... in the emergency department. Whereas I wasn't actually able to apply it myself I was able to understand what they were saying and be like... oh that's cool I learned about that like... and they actually used this here. So no I knew what I learned about is actually gonna be used. So that would motivate me to keep on scribing so then I could umm have that information.. more information like reiterated. And like, I would further my... I don't know like... knowledge
Exposure to physician differential/diagnostic thought processes (3/5)	I think it's definitely directed me more towards... in terms of wanting to become a physician... just because I think it's so ummm it's so mind blowing how much knowledge they have in their head and they can just think of it and I feel any situation they can present they might not know the diagnosis right on the spot, it's so interesting how they solve problems and how they think in order to get to the end point. And I really liked that about it. It's like a puzzle in a sense and I felt that was a really interesting aspect of the profession. OR Ummm yesterday there was someone that came in and they had ruptured their patellar tendon. Andd the doctor just immediately knew, like, okay... this is the test to do. He couldn't lift his leg. This is, like, I know this is a patellar tendon rupture. I thought that was really cool just be able to diagnose and know that muscle injury. Like, the event was super non-specific. Like he'd just fallen, cut his leg. Lke to be able to know that and to go into that detail and just from so much experience. I think that was really cool.
Professionalism development (3/5)	Ummm, I definitely feel like, ethically, there was lots of things that happened that I would want to tell my, like, my friends, my girlfriend. But then I was like, maybe they don't want... like that person wouldn't want me telling what is going on with their lives. Even if I don't say their name, like, they might like see someone with the same kind of issue and be like "Oh its this person and now I know they got this, this, and this wrong with them" and so yeah, and then I feel like... like that could work further through my life. Like, like keep things to myself more. And like yeah. Even if I’m not like, like even if i think of this kind of way, that could get back around. There could be like the information that I learn from it and like know I shouldn't say it to someone else. Yeah OR It was honestly walking into every room you'd be introduced, you'd have to introduce yourself afterwards to the patients. I think that I was held to the same standard as the doctor was. There wasn't really any specific moment where I was like "oh crap" I need to be super professional. It was just the whole experience you had to be really on your game. Like really grown up *laugh*
Improve verbal and non-verbal communication skills/ filtering patient histories between relevant and irrelevant information (2/5)	ummm kind of at first it was hard to ummm pick apart what bits and pieces you need to actually write into the like guidelines. Because a lot of the patients would ramble on about their whole life story and why they’re at this hospital and not the other. Ummm and then and so that was hard at the beginning especially. But then as I went through by the fourth one I was able to pick out what was needed and so yeah. …. Yes, ya. LIke interpreting those signals. Not even…. the doctor would…. more than what the doctor was just saying. Reading his body language. being able to interpret the patients body language. Trying to figure out the gaps that weren't being said necesarily.. in ther interactions.
Perceived value to health care team (5/5)	
Liability (5/5)	
Slowing down physician (5/5).	
slow typing speed (1/5)	if for example I couldn't type things fast enough, like, afterwards we'd have to take the time to go through everything and it would take even more time at the end.
difficulty understanding physician (2/5)	And I'd be like "Okay, can you spell that for me?" and it would be like a word that... just regular word. It wouldn't be like a medical term. But I couldn't hear it. Like he'd have to really slow down. And I'd have to listen to what he was saying. Ummm that was probably the biggest challenge. Just making sure I understood him. And he understood me. But other than that, it was good.
incorrect spelling (4/5) + lack on knowledge of medical terminology (4/5)	I think some of the challenges were the spelling of the common drugs or different anatomy and things like that and diagnoses. I think that those would be things that would improve if I was to do it for longer and I was just to, you know, you kind of learn how to spell complicated drugs just by typing them in regularly. Even with preparing for the project, I found that in the moment if you're typing it and it's so fast paced, you might get the spelling wrong even if you've seen it before but towards the end I did feel like I was spelling words more correctly and I was remembering things from the previous sessions. I would say the spelling was the biggest challenge from the study just because I don't have that much knowledge of pharmaceuticals and that kind of thing at this point but I feel like that would come with time if I was to continue doing it.
direct feedback from physician that scribing decreased efficiency (2/5).	I think she thought for some of [the patients] just because of the short time it was a little inefficient because I think she could've done it faster than I could've. Especially after we would leave the examination room and she'd just look over the things and we'd add things and we'd discuss a little more about what happened.
Asset (5/5)	
Helpful in Psych Consults (1/5)	But one time... there were a few instances where I know she really appreciated the scribing. Those instances specifically were when there were psych consults because there was so much history and there were so many aspects of the story, I think it would've been hard for her to remember every detail going back on it after and inputting it herself. So I think those were the most effective scribing experience for sure and... those specific ones - for the psych consults - I do think that some of the things... even though she could've done it faster herself at that point. I think that if you did it consecutively for a month, I think that you would form more of a team with the doctor... you'd be able to pick up more of just they're manneurisms and things like that ummm that they do during their experiences. And you'd just know to write it down even if they didn't say. So when they review it they wouldn't be adding so many things just because you'd be able to pick up on it more. You'd get used to their style. But I think most of the experience was either neutral or good. I don't think there were many negative experiences.
Increased motivation to pursue medicine, work harder in school (5/5)	
enjoyment and excitement of being in the ED (2/5)	I feel like it really opened my eyes to how interesting and exciting emergency medicine can be. I... because I'm in my undergrad, I feel like there's so much I don't know about the different types of being a doctor and of course when you think about an emergency room doctor you know there's gonna be ummm a variety of things they could see on a daily basis but it really put into perspective ummm the... how interesting it was and uhh kinda the excitability of it all.. I don't know if that makes sense, there's always something new to look at and new to see. There's always something very interesting and fast paced and I kinda like that about it. Just because I enjoyed the experience so much it's going to motivate me to work harder in school and that kind of thing because it seems like such a good career and interesting profession and I definitely want to pursue something like that.. yaa
Demystifying the profession (4/5)	Umm, ya it definitely motivates me to be in medicine more. Because before, as I've said in a previous question. I've had no clue. Now I actually have an idea what it's kinda like. Like, I know it's only in one department. But... Knowing what it is, it's a lot more... it seems like I actually have a proper goal. Rather than I'm just going towards this thing that, that i'm just told is a great thing. Which generally isn't a good way to... like progress. So ya, I think it helped a lot.OR ummmmm. hmm. I'd say it definitely helped with... cause before I was kinda like ohhh I don't really know if I could have the marks or whatever, or even like put in the effort to write my MCAT's and study for them but, like I think being a scribe made me realize that, you know... like I don't know how to say it... That I could do it almost. Kind of. I don't know. It's hard to explain *laugh. But it definitely did boost my self confidence with that stuff so... So seeing other like, the doctors and stuff. And do... watching everything they do and stuff like that made me more interested and kinda boosted my self confidence and I was like OK, maybe I could do it.
Medical School application Strengthened (5/5)	
Character building experiences (4/5)	
Overcoming Adversity/ building empathy (3/5)	the first patient, they had a broken nose and they were… they were in rough shape and umm... anyways I remember I almost passed out after the experience and not because of the sight of the patient or anything like that but it was more so the thought of how much pain the patient was in and having to reset the broken nose after it was already set as the doctor was explaining. That kind of set me off a little bit and it made me a little bit uneasy, but I feel like now that I have that experience and after that I saw many broken bones and it didn't bother me at all. But just to have that initial experience and to have that reaction of... WOW this is real life, this isn't in a movie. People are actually hurt and sick in here. That definitely put in perspective how lucky my health is and things like that, that I don't have to go through those kinds of painful situations in my current status quo - I guess - situation. I'm in pretty good health. I feel like just having that exposure to how real those situations are and how it's directly affecting the patients. It just took me by surprise at the beginning. I was able to process that and add it to my knowledge that... these doctors are really doing important things and... because of that, the pain and seeing people in pain hasn't bothered me as much because I know the doctors are doing things to make it better.
Medical scribing included in resume (3/5)	Yes. absolutely. I think that'll be something that they'll see on my resume that i've written when I apply and they'll be like "Oh.. what.. .no way!"
Scribing Preferred to all other volunteer Experience (5/5)	Well I volunteered before at the hospital. But I found the volunteer capacities they have there are more... bring a patient somewhere.. or like answer some questions that people have. It's not really direct exposure to what doctors are doing. It's more clerical work almost. And, like, that is good to know. Because you are going to have to do that at some point. But it's not the direct exposure that I wanted. Any volunteer things I've done so far haven't been direct exposure so far. So, this was awesome.
Direct exposure to medicine (3/5)	Well I volunteered before at the hospital. But I found the volunteer capacities they have there are more... bring a patient somewhere, or like answer some questions that people have. It's not really direct exposure to what doctors are doing. It's more clerical work almost. And, like, that is good to know. Because you are going to have to do that at some point. But it's not the direct exposure that I wanted. Any volunteer things I've done so far haven't been direct exposure so far. So, this was awesome.
Better learning Experience (2/5)	Okay, so... Ummm coaching for instance. Coaching you're in a more of a leadership role. You're kind of interacting with a different age group. You're interacting with different people. Ummm, and you have to kind of direct more. Whereas this experience with medical scribing. It was... being able to like.. problem solve. Pick a cue. Still interact with people. But you're in a different position. Not one of authority but one of learning. And I thought that was super helpful because... obviously you always want to be learning, and... you always want to be like.. expanding your horizons. Whereas with coaching, it's kind of like, what you know. And you're still expanding on that. But it's more you're directing whereas with the medical scribe you're being directed as to doing something new. If that makes sense.
Excitement (1/5)	I feel like I would probably choose... the scribing experience for the sole purpose that I think although I enjoyed both works - my office research positions and the scribing- I think the scribing was always new and exciting and interesting so it was just a very, it was a very good experience
Greater Impact (1/5)	I definitely prefer the scribing experience mainly because umm I did... there was even though I had mentioned umm there were a few times I didn't have a role in the scribing experience because the physician said just wait. Most of the time there was something for me to do. And I knew this is exactly what I need to do. So do it whereas in the nursing home it was more just like kind of walk around... and like see what people might need.. and like maybe like do it or ask someone. So a lot of the time in the nursing home I kind of walked around but like there was already a bunch of people like who were like trained and older and like actually worked there already helping out these old people with whatever they needed and there was like no one else really to help out.
Better match to career aspirations (1/5)	I think in terms of .. in terms of what I think would look best for me and what I want to do in the future, I would choose the scribing experience. Just because I think it would be more... it looks better almost. Because it is towards what I want to do. Whereas that is just kinda like fun. You know, recreational.
Medical Students	
Learning Experience (5/5)	
Charting skills improved (5/5)	I would say there were some differences. I would say the main difference is that when you're doing the notes. You actually had to think about things a little bit more systematically. But I also found that when I was doing the notes I was doing less thinking. Like I was seeing everything laid out more systematically and discussing it systematically but I felt like I myself was doing less thinking about it because I was just typing everything out verbatim as they said it. And then afterwards the doctor would just go through the note and then correct it. Um and then add things in. Whereas during the shadowing shifts I found that you're doing more independent thinking on your own to figure out why they're asking what they're asking. And figuring out why they're ordering certain things. Like there was more independent thinking because the focus was more on umm watching the consultation vs typing out the notes. That was the main difference between them. But umm yeah so in terms of skill development I would say that during the shadowing you're more focused on critical thinking whereas during the note taking you're more focused on I guess ummm like seeing everything laid out systematically like in order and in steps. OR And I think that though they may try to... uhhh have us do case writeups and that kind of thing in clinical skills. Scribing in the ER is a really good fast way to learn how to come up with basically a case writeup that's a little less formal and what you'd actually be doing in the ER. So I .. I would not be opposed to it being integrated into the curriculum. Or So you've got your HPI and you've got your PMH, social, and then it's also how to write the exam and the procedural notes. Which we aren't taught as much. We're taught the exam but... procedural notes we've never done. So far. So I think it just furthered my ability to chart appropriately. OR The one thing I thought was really useful. Because I'm basically doing clin skills except it's someone else asking. The physician was asking questions I wouldn't think about asking. Which was incredibly useful. So I'd say that was a big positive. It was just like getting another kind of like set of ummm... another person doing the same kind of process that you would do and seeing maybe I should ask this question next time I'm asking someone if.. they have a .. I don't know... thyroid issue for example OR I think that... it would've helped. Ummm, just seeing what other physicians are finding important to write down. Ummm and seeing exactly like how they would like it. Because sometimes they would come check out what I wrote down and be like... "oh well I actually like.. you wrote down word for word what I said but if you put it this way this is how I would, like, write it" So like kinda seeing the difference between what I'm doing now vs what a physician would do.
Scribing and Shadowing complementary learning experiences (4/5)	I will present my patient and my exam to [physician] and then we'll go in again and he'll present... or he'll speak with the patient. Ask some other questions, and he'll do the exam as well. So... I think either way works. One way I'm doing it myself and then when I'm scribing, I'm watching him do it. And I learn both ways. That's how I learn. I like to see things done and I like to do them myself. So it fits my learning style. It's definitely something, like, I wish this was a thing last year. When I was in first year. That would have been really beneficial. I think.
Scribing focuses on pertinent negatives (1/5)	I will present my patient and my exam to [physician] and then we'll go in again and he'll present... or he'll speak with the patient. Ask some other questions, and he'll do the exam as well. So... I think either way works. One way I'm doing it myself and then when I'm scribing, I'm watching him do it. And I learn both ways. That's how I learn. I like to see things done and I like to do them myself. So it fits my learning style. It's definitely something, like, I wish this was a thing last year. When I was in first year. That would have been really beneficial. I think.
Improved ability to come up with differentials (3/5)	ummm I think... it was, it was definitely a good experience to be in the hospital and more clinical experience is always valuable and I think even becoming umm just by recording everything and all the symptoms and history and everything. My physician would have me, kind of in the problems list we would think up differentials and things like that, so I guess it kind of helped me to have a straight forward train of thought where it's like these are my notes and this is why this is on our differential and later on the physician would add to the diagnosis part. So that's good
History Taking Skills (3/5)	Scribing: The one thing I thought was really useful. Because I'm basically doing clin skills except it's someone else asking. The physician was asking questions I wouldn't think about asking. Which was incredibly useful. So I'd say that was a big positive. It was just like getting another kind of like set of ummm... another person doing the same kind of process that you would do and seeing maybe I should ask this question next time I'm asking someone if.. they have a .. I don't know... thyroid issue for example Shadowing: I thought it was much more because I got to actually... i got to ask the questions myself. In the scribing I was obviously just kind of like looking on. Whereas the non-scribing experience I got to see patients and I think obviously when you're seeing patients by yourself and putting those things that you use into action I think you get more out of it. And then... ummm.. it was.. it was a good supplement I think. It was a different experience I think completely. But it wasn't diferent bad. It was just.. I don't know .. different.
Better quality of feedback (1/5)	[during scribing] you're kind of just trying to get all the information down. And if you're lucky the physician explains to you after why they asked these questions. But if they didn't then you're kind of like "Oh... well I’m not sure why they asked this specific question that I didn’t' really learn before". But uhhh ya, um like... whereas in the non-scribing election portion. If you missed something or if you asked something really good, the physician would say "why did you miss this? You should include this; here's why". Or "Good job, that you included that. Because it's super important" for example
I3 training (1/5)	Because if we go into the hospital without knowing how to use the system and what things need to be written down and stuff. It can be very intimidating to be in the hospital the first time practicing medicine as well as trying to learn how to use the system. So i'd say yeah
Interview Directing Skills (1/5)	And in terms of an observational uhhh preparation, like... I am terrible at cutting people off. I'm terrible at redirecting them when they're telling me something that's really not important. But I don't know, I don't feel comfortable cutting them off or redirecting them. And it was, helpful for me to watch Dr. [insert doctor name] do that a certain way. And then another resident do that a certain way. So just seeing how to politely navigate the patient-physician interaction as, like, just a 3rd party observer in the room, or scribe rather, was helpful I think for my future practice. …… [during a shadowing experience] it isn't something I would normally see unless I were scribing or shadowing. And as an elective student in the emergency room. You don't shadow very often. You get a chart and go see a patient.
Shadowing or Scribing preferred	
Shadowing Preferred (4/5)	
More valuable teaching moments during shadowing (1/5)	Ummm I think I preferred the shadowing just because there was more teaching involved. So they would teach me a lot more about what conditions were going on. Their thought processes. Whereas the scribing was more just, keeping up with what they were doing. And not so much the communication about what was going on with the patient. Like, yeah. Like the teaching part. EXAMPLE Okay, ummm so with the shadowing experience we had umm a patient who had ingested a lot of their medication. And we had an idea about what medications it were.. they.. he .. he blah he ingested but ummm wanted to make sure that it wasn't something else. So she actually like taught me how to do.. calculate an anion gap and then showed me his chart and allowed me to go through and calculate the anion gap. To make sure that he was within the range of the medications that he told use he took. And i don't think I would've the gotten time taken out for the doctor to like sit me down and give me that work to do if I was just scribing
Belief that physician perceives students as administrative staff instead of medical students (2/5)	I think it's just if I was in there shadowing you're literally just standing there waiting to get involved and I think the physicians are more inclined to be like "Oh yes, come do this." Like "come have a look at this". Whereas if you're typing the mindset the mindset is you're doing something so I'm just going to let you do that so we're not going to bother you. So I don't know and it's just... I feel like during the physical... when they start doing the physical exam. Yes, you scribe that too but it's also something you can scribe when you leave the room. So even maybe just like having physicians be aware of that. They don't need to NOT BOTHER the scribe. The scribe can still scribe and be interactive with the patient i think, yeah.
Scribing Preferred (1/5)	I think on an actual learning and that sort of basis I actually did prefer the scribing just because I found shadowing... it's interesting, but I find during the history taking sometimes I just find myself not paying attention as much because there's not really a need for me to be paying attention. When you're shadowing you're there just for your own learnign. During physical exams I find myself focused because that's something that's intersting. It's new. Whereas histories, they are very important, sometimes I don't listen. Whereas in the scribing you really had to focus. I think having that you need to focus and pay attention I retained a lot more. And the differentials and all that made a lot more sense putting together a story with an actual diagnosis in the end. Yeah

## References

[1] Babbott S, Manwell LB, Brown R (2014). Electronic medical records and physician stress in primary care: results from the MEMO Study. J Am Med Informatics Assoc.

[2] Heaton HA, Castaneda-Guarderas A, Trotter ER, Erwin PJ, Bellolio MF (2016). Effect of scribes on patient throughput, revenue, and patient and provider satisfaction: a systematic review and meta-analysis. Am J Emerg Med.

[3] Martel ML, Imdieke BH, Holm KM (2018). Developing a medical scribe program at an academic hospital: the Hennepin County medical center experience. Jt Comm J Qual Patient Saf.

[4] Furukawa MF (2011). Electronic medical records and the efficiency of hospital emergency departments. Med Care Res Rev.

[5] Lowry JE (2017). Medical students’ perspectives on their experiences as medical scribes. Medical Students’ Perspectives On Their Experiences As Medical Scribes.

[6] Gellert GA, Ramirez R, Webster SL (2015). The rise of the medical scribe industry. JAMA.

[7] (2019). ScribeAmerica: Medical Scribe. https://www.wayup.com/i-Hospital-and-Health-Care-j-Scribe-ScribeAmerica-992906421/.

[8] (2019). Complete your typing test. https://www.typingtest.com/test.html.

[9] Allen R (2017). Student experience survey questions. Student Experience Survey questions.

[10] Avegno JL, Murphy-lavoie H, Lofaso DP, Moreno-walton L (2012). Medical students’ perceptions of an emergency medicine clerkship. Int J Emerg Med.

[11] Braun V, Clarke V (2006). Using thematic analysis in psychology. Qual Res Psychol.

[12] Millan LR, Azevedo RS, Rossi E, De Marco OLN, Millan MPB, de Arruda PCV (2005). What is behind a student’s choice for becoming a doctor?. Clinics (Sao Paulo).

[13] Kusurkar RA, Croiset G, Galindo-Garré F, Ten Cate O (2013). Motivational profiles of medical students: association with study effort, academic performance and exhaustion. BMC Med Educ.

[14] West CP, Dyrbye LN, Satele D V, Sloan JA, Shanafelt TD (2012). Concurrent validity of single-item measures of emotional exhaustion and depersonalization in burnout assessment. J Gen Intern Med.

[15] Graves PS, Graves SR, Minhas T, Lewinson RE, Vallerand IA, Lewinson RT (2018). Effects of medical scribes on physician productivity in a Canadian emergency department: a pilot study. CMAJ Open.

